# Prevalence of Healthcare Barriers Among US Adults With Chronic Liver Disease Compared to Other Chronic Diseases

**DOI:** 10.1016/j.gastha.2024.05.004

**Published:** 2024-05-17

**Authors:** Carrie R. Wong, Catherine M. Crespi, Beth Glenn, Folasade P. May, Steven-Huy B. Han, Roshan Bastani, James A. Macinko

**Affiliations:** 1 Vatche and Tamar Manoukian Division of Digestive Diseases, Department of Medicine, University of California, Los Angeles, Los Angeles, California; 2 Kaiser Permanente Center for Health Equity, University of California, Los Angeles, Los Angeles, California; 3 Jonsson Comprehensive Cancer Center, University of California, Los Angeles, Los Angeles, California; 4 Department of Health Policy and Management, Fielding School of Public Health, University of California, Los Angeles, Los Angeles, California; 5 Department of Biostatistics, Fielding School of Public Health, University of California, Los Angeles, Los Angeles, California; 6 Department of Community Health Sciences, Fielding School of Public Health, University of California, Los Angeles, Los Angeles, California

**Keywords:** Access to Care, Health Policy, Disparities, Socioeconomic

## Abstract

**Background and Aims:**

The extent of healthcare barriers and its association with acute care use among adults with chronic liver disease (CLD) relative to other chronic conditions remains understudied. We compared the probability of barriers and recurrent acute care use among persons with CLD and persons with chronic obstructive pulmonary disease (COPD) and/or cardiovascular disease (CVD).

**Methods:**

We assembled a population-based, cross-sectional study using pooled self-reported National Health Interview Survey data (2011–2017) among community-dwelling persons. Probability of barriers by disease group (CLD vs COPD/CVD) was assessed using hurdle negative binomial regression.

**Results:**

The sample included 47,037 adults (5062 with CLD, 41,975 with COPD/CVD). The CLD group was younger (median age 55 vs 62 years) and included more Hispanics (17.5% vs 8.6%) and persons with poverty (20.1% vs 15.3%) than the COPD/CVD group. More respondents with CLD vs COPD/CVD reported barriers (44.7% vs 34.4%), including unaffordability (27.5% vs 18.8%), transportation-related (6.1% vs 4.1%), and organizational barriers at entry to (17.6% vs 13.0%) and within healthcare (19.5% vs 14.2%). While adults with CLD were more likely to experience at least 1 barrier (adjusted incident rate ratio, 1.12 [1.01–1.24], *P* = .03), they were not associated with more (1.05 [1.00–2.71], *P* = .06). Probability of recurrent acute care use was associated with more healthcare barriers.

**Conclusion:**

Findings from this nationally representative sample of over 43 million US adults reveal that persons with CLD have increased probability of healthcare barriers, likely related to their higher prevalence of socioeconomic vulnerabilities compared to persons with COPD/CVD. CLD warrants attention as a priority condition in public policies that direct resources towards high-risk populations.

## Graphical abstract

**Figure:**
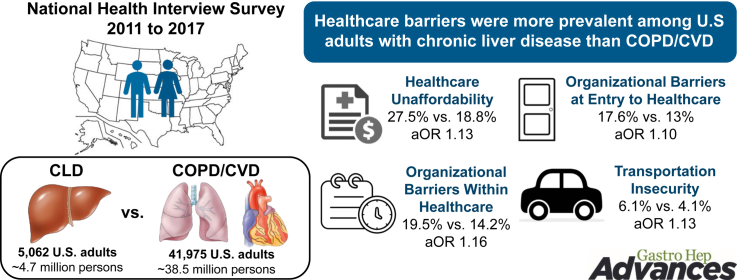

## Introduction

Chronic liver disease (CLD) has become the ninth leading specific cause of death in the United States (US)^1^ and contributes to significant morbidity and mortality, yet it remains under-recognized in public programs compared to other chronic diseases with similar complexity and risk of acute care utilization and mortality. Cardiovascular disease (CVD) and chronic obstructive pulmonary disease (COPD) along with CLD are primary causes of hospital readmissions and deaths in the US.^1^, ^2^, ^3^, ^4^, ^5^ To reduce early hospital readmissions, the Centers for Medicare and Medicaid Services has implemented programs like the Hospital Readmissions Reduction Program (HRRP) for high-risk conditions including COPD and CVD.^6^, ^7^, ^8^ No similar programs exist for persons with CLD despite the dramatic rise in liver-related mortality^9^ and hospitalization rates over the past 2 decades.^5^^,^^10^

Presence and extent of healthcare barriers have been linked to greater risk of acute care use in prior literature.^11^, ^12^, ^13^, ^14^, ^15^ There have been increasing efforts to understand how barriers, such as healthcare affordability and access, influence outcomes for persons with CLD.^16^, ^17^, ^18^, ^19^, ^20^, ^21^, ^22^ Prior studies have found that lack of specialty care, healthcare unaffordability, and transportation insecurity are associated with acute care use and mortality among persons with CLD.^16^, ^17^, ^18^, ^19^, ^20^, ^21^ Less is known about the prevalence of different types of healthcare barriers across the care seeking continuum, including organizational barriers at the point of entry to and barriers within the healthcare system. The relative influence of various population characteristics, such as socioeconomic circumstances, on the likelihood of encountering healthcare barriers among CLD patients also remains unexplored.

To our knowledge, the extent of barriers to care and the relationship between the prevalence of healthcare barriers and recurrent acute care use among US adults with CLD compared to other serious chronic diseases remains unknown. Therefore, we analyzed data from the National Health Interview Survey (NHIS) to compare self-reported healthcare barriers among community-dwelling persons representative of the national population with CLD or COPD and/or CVD.

## Methods

### Data Source

The institutional review board at the University of California, Los Angeles exempted this study from review because it used publicly available deidentified data. The study follows the Strengthening the Reporting of Observational Studies in Epidemiology reporting guideline.

We assembled a pooled cross-sectional dataset using annual NHIS data, from 2011 to 2017, obtained from the Integrated Public Use Microdata Series Health Surveys.^23^ In accordance to NHIS guidance,^24^ we divided the sample weight by the number of years included. NHIS is an annual in-person household interview survey conducted by the US Census Bureau to collect self-reported sociodemographic factors, health, behaviors, and healthcare experiences from civilian, noninstitutionalized persons (eMethods in Supplement).^24^ We used the Sample Adult Core, which had a mean conditional response and final response rate of 80.7% and 60.4%, respectively during the study period.

The study period was selected to start in 2011 to capture healthcare experiences after the enactment of the Affordable Care Act. The study period ended in 2017 because specific healthcare barriers were no longer captured in the NHIS starting in 2018.

### Study Population

We included adult participants at least 18 year old with affirmative responses to questions about CLD (main exposure) and COPD and/or CVD (comparison group) (Figure A1 in Supplement). CLD was defined as answering *yes* to the questions, “Has a doctor or other health professional ever told you that you had any kind of chronic, or long-term liver condition” or “During the past 12 months, have you been told by a doctor or other health professional that you had any kind of liver condition?” as previously done.^19^^,^^21^^,^^25^ Following the CDC definition of chronic obstructive pulmonary disease (COPD)^26^ and similar to prior work,^27^^,^^28^ COPD in this study was defined as answering *yes* to the questions, “Have you ever been told by a doctor or other health professional that you had chronic obstructive pulmonary disease, also called COPD?”, “During the past 12 months, have you been told by a doctor or other health professional that you had chronic bronchitis?”, or “Have you ever been told by a doctor or other health professional that you had emphysema?”. CVD was defined as having an affirmative response to the following questions: “Have you ever been told by a doctor or other health professional that you had… coronary heart disease?”, “… a heart attack?”, “…any kind of heart condition or heart disease”, “… angina”, or “… a stroke?” following the same definition as prior work.^29^

The CLD group included adults with COPD or CVD because concomitant COPD or CVD is common among persons with CLD in the real-world.^30^, ^31^, ^32^ Persons with COPD and CVD were grouped together because of considerable overlap between both disease groups.

### Study Outcomes

#### Healthcare barriers

The primary outcome was the number of healthcare barriers, which was captured via self-reported surveys. We also assessed a binary indicator of any healthcare barriers vs none. Specific healthcare barriers, representing the care seeking continuum, included healthcare unaffordability (foregoing needed medical care, prescription medication, follow-up care, and/or specialty care due to cost), organizational barriers at the point of entry to healthcare (lack of usual place for care, trouble finding a general provider, declined by clinic as a new patient, clinic declined one’s healthcare coverage), organizational barriers within healthcare (could not get an appointment, inconvenient clinic hours, could not reach the clinic by telephone, long wait to see the doctor at the clinic), and delays in medical care related to lack of transportation in the past year (eMethods in Supplement). Specific healthcare barriers were also examined by type (healthcare unaffordability, organizational barrier at entry to healthcare, organizational barrier within healthcare, transportation insecurity) vs none.

#### Recurrent acute care use

A secondary outcome was recurrent acute care use. We defined recurrent acute care use as having at least 2 emergency department (ED) visits and/or overnight hospital admissions in the past year. ED visits were included because approximately 40% of recurrent acute care encounters within 30 days of a hospital discharge can occur in the ED.^33^

#### Covariates

Covariates included the respondents’ self-reported age (18–34, 35–54, 55–65, 65–85 year old), sex, race or ethnicity (Non-Hispanic [NH] White, Hispanic, NH Black, NH Asian, NH American Indian or Alaskan Native, NH Other), number of comorbidities, functional limitation due to health, fair or poor health status, education (less or greater than high school graduate level), household support (ie, living alone or not), employment, household poverty (less than or at least 200% of the federal poverty level), receipt of government support for income, housing, food, or other welfare, insurance coverage (none, public, private, Medicare), survey period (2011–2013, 2014–2017), and US Census region (Northeast, North Central/Midwest, South, West). We controlled for these covariates based on their hypothesized association with the outcomes (eMethods in Supplement).

### Statistical Analysis

Each annual survey was appended to create a pooled dataset. Survey weights were applied using the Stata –*svy*- command or person weights with clustering at the primary sampling unit.

We compared sociodemographic and health characteristics of respondents in the CLD and COPD/CVD groups using adjusted Wald and Chi-squared tests for continuous and categorical variables, respectively. We also assessed relationships between the covariates and outcomes by disease group.

The number of healthcare barriers (primary outcome) was modeled using a 2-part hurdle logit-negative binomial regression model based on fit statistics for different count models and model assumptions (eMethods and Table A1 in Supplement). Logistic regression analysis was used for the outcome of any barriers and specific healthcare barriers. Covariates were included in multivariable regression analyses based on their different frequencies between the CLD and COPD/CVD disease groups (Tables A2 and A3 in Supplement) and results of our nested hurdle logit-negative binomial regression models (Table A4 and eMethods in Supplement).

Stratified analyses were performed using logistic regression to estimate the probability of any barriers to care within age categories (18–34, 35–54, 55–64, at least 65 year old), sex, race or ethnicity (Hispanic or NH White, Black/African-American, Asian, American Indian/Alaskan Native, or Other), receipt of government support, and insurance (none, public, Medicare, private). Adjustment for additional covariates was avoided in the stratified analysis to provide a more nuanced assessment of where disparities may exist by the specific aforementioned categories. As a sensitivity analysis, we repeated the main analysis using only respondents with CLD without any concomitant COPD or CVD vs COPD/CVD (without CLD).

We evaluated the association between recurrent acute care use (secondary outcome) and the prevalence of healthcare barriers by disease group using multivariable logistic regression.

Probabilities were obtained using predictive margins after obtaining estimates from the regression models for both the primary and secondary outcomes.^34^ Statistical significance was defined as a 2-sided *P* value of less than .05 for all analyses. All analyses were performed using Stata SE version 18.0 (StataCorp).

## Results

### Study Population Characteristics

The final sample included 47,037 adults, which provided weighted estimates for 43,264,685 persons. The CLD group consisted of 5062 adults (representative of 4,742,444 persons), and the COPD/CVD (non-CLD group) included 41,975 persons (representative of 38,522,241 adults). The CLD group included 1422 respondents with concomitant CVD (28.1%; 95% confidence interval [CI], 26.5%–29.9%) and 881 adults with COPD (17.4%; 95% CI, 16.1%–18.8%), which reflected a more accurate representation of the liver disease population in clinical settings.^30^, ^31^, ^32^

The CLD group was younger (median age [range], 55 [18–85] vs 62 [18–85] years) and had more individuals who identified as Hispanic (+8.9 percentage points [pp]; 95% CI, 7.4%–10.4%; *P* < .001) and reported having fair or poor health (+8.1 pp; 95% CI, 6.2%–10.0%; *P* < .001) compared to the COPD/CVD group. While more persons with CLD were employed (+6.1 pp; 95% CI, 4.2%–8.0%; *P* < .001), the CLD group had more household poverty (+4.8 pp; 95% CI, 3.4%–6.2%; *P* < .001), government support (+8.0 pp; 95% CI, 6.3%–9.7%; *P* < .001), and uninsurance (+3.3 pp; 95% CI, 2.0%–4.5%; *P* < .001) or public insurance (+7.9 pp; 95% CI, 6.2%–9.5%; *P* < .001) compared to the COPD/CVD group. Additional characteristics are provided in Table 1.

**Table 1:** Sociodemographic and Health Characteristics, by Chronic Liver Disease and Chronic Obstructive Pulmonary Disease and/or Cardiovascular Disease (N = 47,037)

Characteristic	Total sample	Chronic liver disease^a^	COPD and/or CVD^b^	*P* value
Respondents, unweighted no.	47,037	5062	41,975	
Estimated population, weighted no.	43,264,685	4,742,444	38,522,241	
Age groups, y^c^				
18–34	10.8 (10.4–11.3)	13.3 (12.0–14.6)	10.5 (10.0–11.1)	<.001
35–54	24.8 (24.3–25.4)	35.5 (33.8–37.2)	23.5 (23.0–24.1)	
55–64	22.9 (22.4–23.5)	28.8 (27.2–30.4)	22.2 (21.7–22.8)	
65–85	41.4 (40.7–42.1)	22.5 (21.0–24.1)	43.7 (43.0–44.5)	
Female sex	51.8 (51.1–52.4)	51.5 (49.6–53.3)	51.8 (51.1–52.5)	.73
Race or ethnicity^d^				<.001
White	74.3 (73.5–75.0)	65.8 (64.0–67.6)	75.3 (74.5–76.1)	
Black or African American	10.8 (10.3–11.3)	8.4 (7.5–9.3)	11.1 (10.6–11.7)	
Hispanic	9.6 (9.1–10.1)	17.5 (16.0–19.1)	8.6 (8.1–9.1)	
Asian	2.9 (2.7–3.1)	5.0 (4.2–5.9)	2.6 (2.4–2.9)	
American Indian or Alaska Native	0.7 (0.6–0.9)	1.0 (0.7–1.5)	0.7 (0.5–0.8)	
Other	1.8 (1.6–1.9)	2.3 (1.8–2.9)	1.7 (1.5–1.9)	
Comorbidity burden				
Number of comorbidities, median (range)^e^	3 (1–10)	3 (1–10)	3 (1–9)	<.001
Functional limitation due to health^f^ (n = 46,933)	68.1 (67.4–68.8)	68.2 (66.4–69.8)	68.1 (67.4–68.8)	.95
Fair or poor health^g^ (n = 47,009)	34.2 (33.6–34.8)	41.4 (39.7–43.2)	33.3 (32.6–34.0)	<.001
Education attainment (n = 46,792)				.13
Less than high school graduate level	6.6 (6.2–6.9)	7.2 (6.3–8.2)	6.5 (6.1–6.8)	
Employment (n = 46,950)				<.001
Currently unemployed	63.2 (62.5–63.9)	57.8 (55.9–59.6)	63.9 (63.1–64.6)	
Living alone	24.5 (23.9–25.0)	23.2 (22.0–24.5)	24.6 (24.1–25.2)	.04
Household income^h^ (n = 43,616)				<.001
Below poverty threshold	15.8 (15.3–16.3)	20.1 (18.7–21.5)	15.3 (14.7–15.8)	
Receipt of any government support^i^	22.4 (21.7–23.0)	29.5 (27.9–31.1)	21.5 (20.8–22.2)	<.001
Income support^j^ (n = 46,957)	7.5 (7.2–7.8)	11.1 (10.1–12.2)	7.0 (6.7–7.4)	<.001
Rent assistance^k^ (n = 46,951)	5.4 (5.1–5.8)	6.3 (5.6–7.1)	5.3 (5.0–5.7)	.01
Food support^l^ (n = 46,976)	18.3 (17.8–18.9)	24.4 (22.9–26.0)	17.6 (17.0–18.2)	<.001
Other welfare^m^ (n = 46,935)	1.0 (0.9–1.1)	1.7 (1.3–2.2)	0.9 (0.8–1.0)	<.001
US region				<.001
Northeast	17.0 (16.3–17.8)	16.4 (15.0–17.9)	17.1 (16.3–17.9)	
North Central/Midwest	24.3 (23.4–25.1)	19.8 (18.4–21.4)	24.8 (23.9–25.7)	
South	38.5 (37.5–39.5)	36.1 (34.2–38.0)	38.8 (37.8–39.9)	
West	20.2 (19.4–21.0)	27.7 (25.9–29.5)	19.3 (18.5–20.1)	
Insurance coverage^n^ (n = 46,034)				
None	8.3 (7.9–8.7)	11.2 (10.1–12.4)	7.9 (7.5–8.3)	<.001
Public insurance	16.6 (16.1–17.1)	23.6 (22.0–25.2)	15.7 (15.2–16.2)	
Medicare	21.3 (20.7–21.9)	15.3 (13.9–16.7)	22.0 (21.4–22.7)	
Private insurance	53.9 (53.1–54.6)	50.0 (48.1–51.9)	54.3 (53.5–55.1)	

Data are reported as percent values with 95% confidence intervals unless otherwise indicated.

Denominators per disease category are reported as unweighted observations.

Weighted estimates were obtained using *svy*. Categorical variables were compared using Chi-squared tests. The number of comorbidities was compared using an adjusted Wald test and the median (range) were obtained using the sampling weight as an analytic weight.

BMI, body mass index; COPD, chronic obstructive pulmonary disease; CVD, cardiovascular disease; SNAP, Supplemental Nutrition Assistance Program; SSI, Supplemental Security Income; WIC, Special Supplemental Nutrition Program for Women, Infants, and Children.

a Chronic liver disease status is based on self-reported responses about ever having any chronic liver condition and/or any kind of liver condition in the past year.

b COPD, status is based on self-reported responses about ever being diagnosed with COPD, emphysema, and/chronic bronchitis. CVD, status is based on self-reported responses about ever being diagnosed with coronary heart disease, myocardial infarction, heart condition or disease, angina, and/or stroke.

c Age groups include the following years: 18–34, 35–55, 56–64, and 65–85 year old.

d Race or ethnicity includes self-reported Non-Hispanic White (*White*), Non-Hispanic Black (*Black or African-American*), Hispanic (*Hispanic*), Non-Hispanic Asian (*Asian*), Non-Hispanic American Indian or Alaska Native (*American Indian or Alaska Native*), or Non-Hispanic Other (*Other*) which includes multiple race and race groups that are not releasable.

e Comorbidity count includes self-reported arthritis, asthma, BMI ≥30, cancer, CVD, chronic liver disease; COPD, diabetes, hypertension, and kidney disease.

f Functional limitation due to health includes responses about having any functional difficulty because of a health problem.

g Fair or poor health includes responses about self-reported health status and is compared to those with excellent, very good, or good health.

h Poverty threshold is based on family size, number of children under 18 year old, and reported before-tax combined money income from all sources, excluding noncash benefits, during the preceding calendar year as compared to the U.S., Census Bureau’s poverty thresholds for the preceding calendar year. The household income category of *Below poverty threshold* includes persons who report household income below the poverty threshold (vs at or above poverty threshold).

i Receipt of any government support includes receiving any support for income (SSI, and/or cash assistance), housing (rent assistance), food (food stamps, SNAP, and/or WIC), and/or other welfare (assistance with getting a job, placement in education or job training programs, transportation, or childcare) in the previous calendar year.

j Income support includes responses about receiving income from SSI, and/or cash assistance in the previous calendar year.

k Rent assistance includes responses about having received public rent assistance.

l Food support includes responses about receiving any food stamps, SNAP, benefits, and/or WIC.

m Other welfare includes assistance with getting a job, placement in education or job training programs, transportation, or childcare).

n Insurance category *None* includes persons without any insurance coverage including having only single service plans, category *Public insurance* includes any public insurance via Medicaid, other state or local government program, Children’s Health Insurance Program, or Medicare for dual enrollees, category *Medicare* includes Medicare only beneficiaries, and category *Private insurance* includes enrollment in any private insurance.

### Healthcare Barriers

More respondents in the CLD group experienced any barriers to care compared to the COPD/CVD group (+10.3 pp; 95% CI, 8.4%–12.3%; *P* < .001). Adults with CLD also reported higher proportions of all types of barriers, including organizational barriers at the point of entry to healthcare (+4.6 pp; 95% CI, 3.2%–6.1%; *P* < .001), organizational barriers within healthcare (+5.3 pp; CI, 3.8%–6.7%; *P* < .001), financial barriers (+8.7 pp; 95% CI, 7.0%–10.4%; *P* < .001), and lack of transportation (+2.1 pp; 95% CI, 1.2%–2.9%; *P* < .001). Additional data on specific healthcare barriers are in Table 2.

**Table 2:** Healthcare Barriers and Acute Care Utilization, by Chronic Liver Disease and Chronic Obstructive Pulmonary Disease and/or Cardiovascular Disease (N = 47,037)

Characteristic	Total sample	Chronic liver disease^a^	COPD and/or CVD^b^	*P* value
Respondents, unweighted no.	47,037	5062	41,975	
Estimated population, weighted no.	43,264,685	4,742,444	38,522,241	
Any barriers to care^a^	35.5 (34.9–36.1)	44.7 (42.9–46.5)	34.4 (33.7–35.0)	<.001
Number of barriers to care, median (range)^b^	0 (0–13)	0 (0–13)	0 (0–13)	<.001
Financial barrier^c^ (n = 47,036)	19.8 (19.3–20.3)	27.5 (26.0–29.2)	18.8 (18.3–19.4)	<.001
Foregone medical care due to unaffordability (n = 47,019)	10.2 (9.8–10.6)	13.5 (12.2–14.8)	9.8 (9.3–10.2)	<.001
Foregone follow-up care due to unaffordability (n = 46,610)	6.4 (6.1–6.7)	9.5 (8.5–10.5)	6.0 (5.7–6.4)	<.001
Foregone specialty care due to unaffordability (n = 46,609)	7.7 (7.4–8.1)	11.7 (10.5–12.9)	7.3 (6.9–7.6)	<.001
Foregone medication due to unaffordability (n= 46,628)	12.9 (12.5–13.4)	17.8 (16.5–19.2)	12.3 (11.9–12.8)	<.001
Organizational barrier at the entry to healthcare^d^ (n= 46,722)	13.5 (13.1–14.0)	17.6 (16.3–19.1)	13.0 (12.6–13.5)	<.001
Trouble finding a provider (n = 46,671)	4.4 (4.2–4.7)	6.3 (5.4–7.2)	4.2 (3.9–4.4)	<.001
Declined as a new patient (n = 46,648)	3.8 (3.6–4.0)	6.5 (5.6–7.5)	3.5 (3.2–3.7)	<.001
Health coverage declined (n = 46,632)	4.9 (4.6–5.1)	7.1 (6.2–8.0)	4.6 (4.3–4.9)	<.001
No usual place for care (n = 46,718)	6.5 (6.2–6.8)	7.5 (6.6–8.6)	6.4 (6.0–6.7)	.02
Organizational barrier within healthcare^e^ (n = 46,640)	14.8 (14.3–15.3)	19.5 (18.1–20.9)	14.2 (13.7–14.7)	<.001
No appointment soon enough (n = 46,626)	9.0 (8.7–9.4)	12.2 (11.1–13.3)	8.7 (8.3–9.0)	<.001
Inconvenient clinic hours (n = 46,618)	3.9 (3.7–4.1)	4.8 (4.2–5.6)	3.8 (3.5–4.0)	.001
Could not get through by phone (n = 46,634)	4.0 (3.7–4.2)	5.5 (4.8–6.4)	3.8 (3.5–4.0)	<.001
Long waiting time at clinic (n = 46,619)	6.7 (6.4–7.1)	8.8 (7.9–9.9)	6.5 (6.1–6.8)	<.001
Other barrier^f^				
Lack of transportation to receive timely care (n = 46,631)	4.3 (4.0–4.6)	6.1 (5.4–7.0)	4.1 (3.8–4.3)	<.001
Acute care use^g^ (n = 47,023)	39.1 (38.5–39.6)	29.2 (27.7–30.8)	24.0 (23.4–24.5)	<.001
At least 2 hospitalizations (n = 46,941)	7.9 (7.6–8.2)	9.9 (8.9–10.9)	7.6 (7.3–8.0)	<.001
At least 2 ED visits (n = 46,416)	16.2 (15.7–16.6)	21.2 (19.8–22.7)	15.5 (15.1–16.0)	<.001

Data are reported as percent values with 95% confidence intervals unless otherwise indicated.

Denominators per disease category are reported as unweighted observations.

Weighted estimates were obtained using *svy*. Categorical variables were compared using Chi-squared tests The number of barriers to care was compared using an adjusted Wald test and the median (range) were obtained using the sampling weight as an analytic weight.

COPD, chronic obstructive pulmonary disease; CVD, cardiovascular disease; ED, emergency department.

a Any barriers to care includes affirmative responses to questions about needing but foregoing medical care, follow-up, specialty care, and/or prescription medication due to unaffordability, trouble finding a provider, being declined as a new patient, having health coverage declined, not having a usual place for routine or sick care, having delays in medical care because one could not get an appointment soon enough, clinic or doctor’s office was closed, could not get through by phone, had a long wait time to see the doctor, and/or lacked transportation.

b Number of barriers to care includes affirmative responses to questions about needing but foregoing medical care, follow-up, specialty care, and/or prescription medication due to unaffordability, trouble finding a provider, being declined as a new patient, having health coverage declined, not having a usual place for routine or sick care, having delays in medical care because one could not get an appointment soon enough, clinic or doctor’s office was closed, could not get through by phone, had a long wait time to see the doctor, and/or lacked transportation.

c Financial barrier includes affirmative responses to questions about foregoing needed medical care, follow-up, specialty care, and/or prescription medications due to unaffordability in the past 12 months.

d Organizational barrier at the entry to healthcare includes affirmative responses to questions about having trouble finding a provider, being declined as a new patient, having health coverage declined, and/or not having a usual place for routine or sick care.

e Organizational barrier within healthcare includes self-reporting delayed medical care because one could not get an appointment soon enough, clinic or doctor’s office was closed, could not get through by phone, and/or had a long wait time to see the doctor.

f Other barrier includes self-reported lack of transportation to receive timely care.

g Acute care use is defined as at least 2 overnight admissions or emergency department visits in the past year.

**Figure 1:**
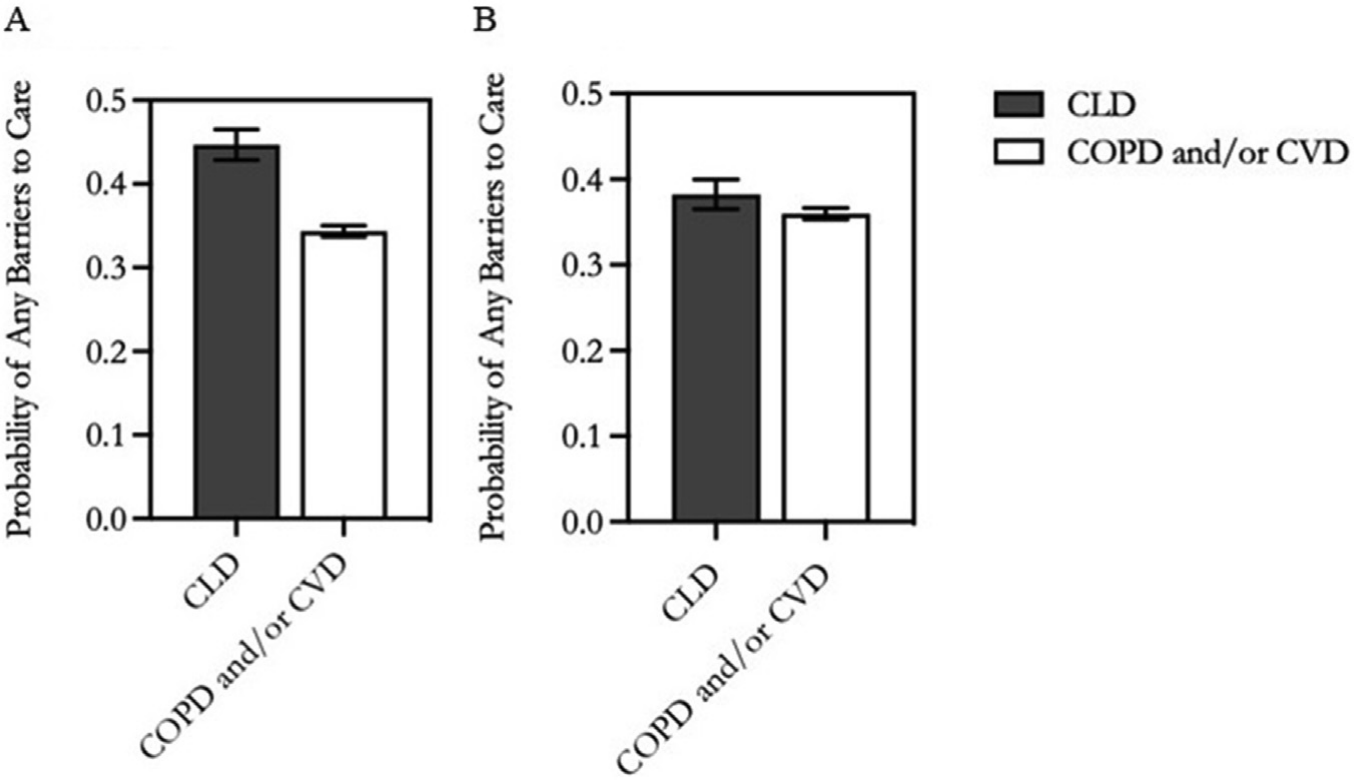
(A) Unadjusted probability of any barriers to care for adults with chronic liver disease compared to chronic obstructive pulmonary disease and/or cardiovascular disease (N = 47,037). (B) Adjusted probability of any barriers to care for adults with chronic liver disease compared to chronic obstructive pulmonary disease and/or cardiovascular disease (n = 42,370). Source: National Health Interview Survey, 2011–2017. Predicted probabilities were obtained from a logistic regression model. The adjusted model included the disease group, sex, age, race or ethnicity, comorbidity count, fair or poor health, functional limitation due to health, education, employment, living alone, household poverty, receipt of government support, insurance, US Census region, and year. CLD, chronic liver disease; COPD, chronic obstructive pulmonary disease; CVD, cardiovascular disease.

The CLD group was 54% more likely to have any barriers to care than the COPD/CVD group in the unadjusted analysis (incident rate ratio [IRR], 1.54; 95% CI, 1.43–1.67; *P* < .001). The adjusted likelihood of having any barriers to care was 12% greater for respondents with CLD compared to those with COPD/CVD (IRR, 1.12; 95% CI, 1.01–1.24; *P* = .03) (Table 3). The unadjusted number of barriers was significantly different between the 2 disease groups (IRR, 1.22; 95% CI, 1.16–1.28; *P* < .001), although the adjusted estimate was not (IRR, 1.05; 95% CI, 1.00–2.71; *P* = .06). For CLD, the predicted probability of any healthcare barriers was 0.45 (95% CI, 0.43–0.47) and 0.38 (95% CI, 0.37–0.40) in the unadjusted and adjusted models, respectively, while these values were 0.34 (95% CI, 0.34–0.35) and 0.36 (95% CI, 0.35–0.37) for COPD/CVD (Figure 1A and B).

**Table 3:** Adjusted Incident Rate Ratios to Assess the Relationship Between Healthcare Barriers and Chronic Liver Disease vs Chronic Obstructive Pulmonary Disease and/or Cardiovascular Disease (n = 42,370)

Characteristic	Any barriers			Number of barriers		
	IRR	95% CI	*P* value	IRR	95% CI	*P* value
CLD (vs COPD/CVD)	1.12	1.01–1.24	.03	1.05	1.00–2.71	.06
Biological female sex	1.12	1.03–1.21	.01	1.22	1.18–3.25	<.001
Age (y) (vs 65 and older)^a^						
18–34	4.51	3.94–5.16	<.001	2.07	1.91–6.74	<.001
35–54	3.03	2.75–3.34	<.001	1.92	1.83–6.24	<.001
55–64	2.13	1.97–2.29	<.001	1.59	1.50–4.48	<.001
Race or ethnicity (vs White)^b^						
Black/African American	0.98	0.92–1.05	.61	0.97	0.92–2.51	.261
Hispanic	1.07	0.97–1.18	.19	1.02	0.93–2.55	.63
Asian	1.05	0.91–1.22	.51	0.86	0.77–2.16	.007
American Indian/Alaskan Native	0.75	0.59–0.95	.02	0.73	0.66–1.94	<.001
Other	1.20	1.04–1.39	.01	1.05	0.98–2.66	.20
Comorbidity count^c^	1.09	1.07–1.10	<.001	1.04	1.03–2.79	<.001
Fair/poor health	1.37	1.30–1.45	<.001	1.21	1.13–3.09	<.001
Functional limitation due to health	1.81	1.64–2.01	<.001	1.47	1.40–4.04	<.001
Less than high school graduate education	0.97	0.91–1.02	.22	0.99	0.93–2.54	.62
Unemployment	0.89	0.83–0.97	.01	0.97	0.92–2.51	.21
Living alone	1.29	1.21–1.37	<.001	1.15	1.12–3.05	<.001
Below federal poverty level	1.08	0.98–1.18	.11	1.09	1.04–2.83	<.001
Receipt of government support^d^	1.56	1.46–1.67	<.001	1.17	1.11–3.04	<.001
Insurance (vs private insurance)^e^						
No insurance	5.50	4.99–6.07	<.001	1.72	1.63–5.11	<.001
Public insurance	1.08	0.94–1.23	.27	1.14	1.06–2.90	<.001
Medicare	1.35	1.26–1.44	<.001	1.25	1.18–3.26	<.001
US region (vs Northeast)						
North Central/Midwest	1.23	1.07–1.40	.003	1.08	1.03–2.80	.001
South	1.26	1.19–1.33	<.001	1.11	1.05–2.87	<.001
West	1.45	1.36–1.55	<.001	1.29	1.23–3.41	<.001
Survey year (vs 2011–2013)						
2014–2017	0.99	0.93–1.06	.86	1.04	1.01–2.74	.02

Weighted estimates were obtained using probability weights (pweight) and clustering at the primary sampling units.

CLD, chronic liver disease; COPD, chronic obstructive pulmonary disease; CVD, cardiovascular disease; BMI, body mass index; IRR, incident rate ratio; SNAP, Supplemental Nutrition Assistance Program; SSI, supplemental security income; WIC, Special Supplemental Nutrition Program for Women, Infants, and Children.

a Age 65 and older is used as the reference group because respondents in both disease groups would become Medicare-eligible.

b Race or ethnicity includes self-reported Non-Hispanic White (*White*), Non-Hispanic Black (*Black or African-American*), Hispanic (*Hispanic*), Non-Hispanic Asian (*Asian*), Non-Hispanic American Indian or Alaska Native (*American Indian or Alaska Native*), or Non-Hispanic Other (*Other*) which includes multiple race and race groups that are not releasable.

c Comorbidity count includes self-reported arthritis, asthma, BMI ≥30, cancer, CVD, chronic liver disease; COPD, diabetes, hypertension, and kidney disease.

d Receipt of any government support includes receiving any support for income (SSI, and/or cash assistance), housing (rent assistance), food (food stamps, SNAP, and/or WIC), and/or other welfare (assistance with getting a job, placement in education or job training programs, transportation, or childcare) in the previous calendar year.

e Insurance category *None* includes persons without any insurance coverage including having only single service plans, category *Public insurance* includes any public insurance via Medicaid, other state or local government program, Children’s Health Insurance Program, or Medicare for dual enrollees, category *Medicare* includes Medicare only beneficiaries, and category *Private insurance* includes enrollment in any private insurance.

**Figure 2:**
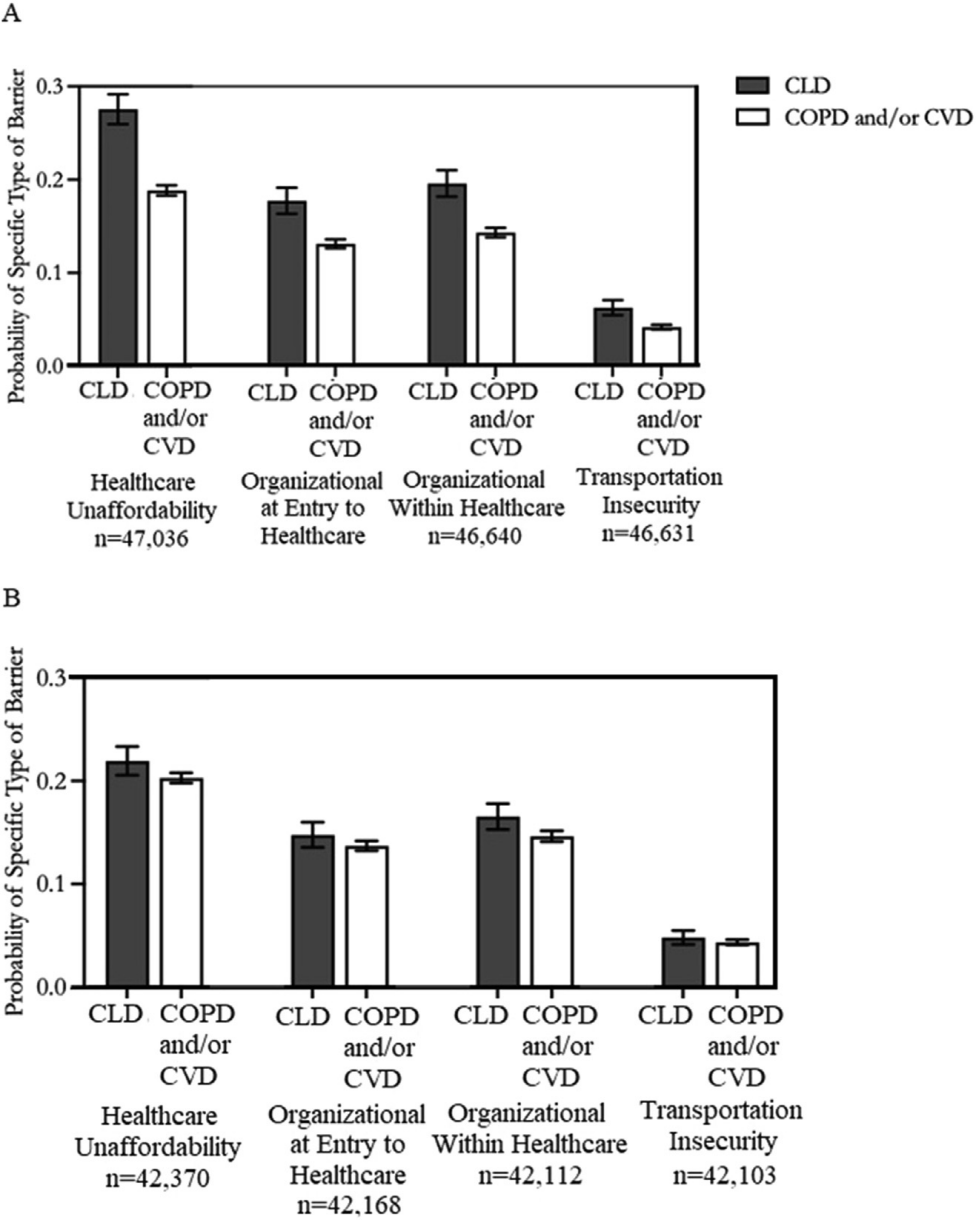
(A) Unadjusted probability of types of healthcare barriers for adults with chronic liver disease compared to chronic obstructive pulmonary disease and/or cardiovascular disease. (B) Adjusted probability of types of healthcare barriers for adults with chronic liver disease compared to chronic obstructive pulmonary disease and/or cardiovascular disease. Source: National Health Interview Survey, 2011–2017. Predicted probabilities were obtained from a logistic rgression model. The adjusted model included the disease group, sex, age, race or ethnicity, comorbidity count, fair or poor health, functional limitation due to health, education, employment, living alone, household poverty, receipt of government support, insurance, US Census region, and year. Unadjusted model estimates for CLD vs COPD/CVD were the following: healthcare unaffordability OR 1.64 (95% CI 1.50–1.79), *P* value <.001; organizational at entry to healthcare OR 1.43 (95% CI 1.29–1.59), *P* value <.001; organizational within healthcare OR 1.46 (95% CI 1.33–1.61), *P* value <.001; transportation insecurity OR 1.55, 95% CI 1.33–1.80, *P* value <.001. Adjusted model estimates for CLD vs COPD/CVD were the following: healthcare unaffordability OR 1.13 (95% CI 1.01–1.27), *P* value .03; organizational at entry to healthcare OR 1.10 (95% CI 0.98–1.24), *P* value .11; organizational within healthcare OR 1.16 (95% CI 1.05–1.28), *P* value .004; transportation insecurity OR 1.13, 95% CI 0.94–1.35), *P* value .19. CI, confidence interval; CLD, chronic liver disease; COPD, chronic obstructive pulmonary disease; CVD, cardiovascular disease; OR, odds ratio.

Respondents with CLD were significantly more likely to have specific healthcare barriers, including healthcare unaffordability, organizational barriers at entry to and within healthcare, and transportation insecurity, compared to individuals with COPD/CVD (Figure 2A). The unadjusted predicted probabilities for any specific type of healthcare barrier for persons with CLD were 0.28 (95% CI, 0.26–0.29), 0.18 (95% CI, 0.16–0.19), 0.19 (0.18–0.21), and 0.06 (0.05–0.07) for healthcare unaffordability, organizational barriers at entry to healthcare, organizational barriers within healthcare, and transportation insecurity, respectively. Unadjusted probabilities for respondents with COPD/CVD were 0.19 (95% CI, 0.18–0.19) for healthcare unaffordability, 0.13 (95% CI, 0.13–0.13) for organizational barriers at entry, 0.14 (95% CI, 0.14–0.15) for organizational barriers within healthcare, and 0.04 (95% CI, 0.04–0.04) for transportation insecurity. After adjusting for covariates, including differences in sociodemographic and insurance characteristics, healthcare unaffordability (odds ratio [OR], 1.13; 95% CI, 1.01–1.27) and organizational barriers within healthcare (OR, 1.16; 95% CI, 1.05–1.28) remained significantly more likely for respondents with CLD to encounter than persons with COPD/CVD (Figure 2B).

**Figure 3:**
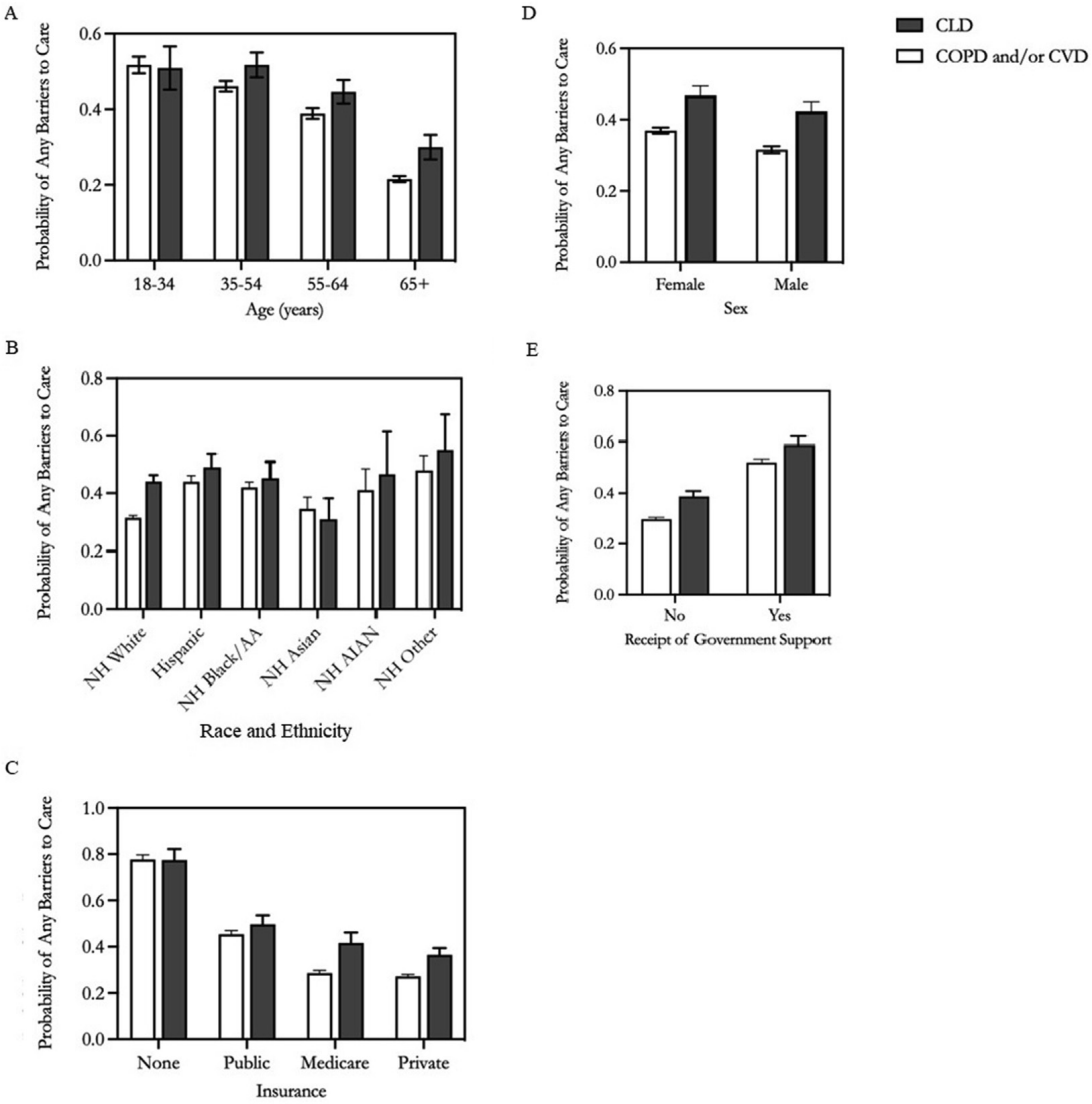
(A) Probability of any healthcare barriers for adults with chronic liver disease compared to chronic obstructive pulmonary disease and/or cardiovascular disease, by age. (B) Probability of any healthcare barriers for adults with chronic liver disease compared to chronic obstructive pulmonary disease and/or cardiovascular disease, by race or ethnicity. (C) Probability of any healthcare barriers for adults with chronic liver disease compared to chronic obstructive pulmonary disease and/or cardiovascular disease, by insurance. (D) Probability of any healthcare barriers for adults with chronic liver disease compared to chronic obstructive pulmonary disease and/or cardiovascular disease, by sex. (E) Probability of any healthcare barriers for adults with chronic liver disease compared to chronic obstructive pulmonary disease and/or cardiovascular disease, by receipt of government support. Source: National Health Interview Survey, 2011–2017. Predicted probabilities were obtained from a logistic regression model that adjusts for disease group and age (A), disease group and race or ethnicity (B), disease group and insurance (C), disease group and sex (D), and disease group and receipt of government support (E). AA, African American, AIAN, American Indian or Alaskan Native; COPD, chronic obstructive pulmonary disease; CLD, chronic liver disease; CVD, cardiovascular disease; NH, Non-Hispanic.

In stratified analyses (Figure 3A–E), most adults with CLD had higher probabilities of any healthcare barriers compared to respondents with COPD/CVD. The difference in predicted probability for any healthcare barriers between CLD and COPD/CVD was largest for adults at least 65 year old (+0.08; 95% CI, 0.05–0.12), who identify as NH White (+0.12; 95% CI, 0.10–0.15), and those who were Medicare enrollees (+0.13; 95% CI, 0.08–0.18). While females (compared to males) and recipients of government support (compared to nonrecipients) had higher predicted probabilities for any healthcare barriers, the difference between respondents with CLD and COPD/CVD were similar.

In sensitivity analysis that compared adults with only CLD without concomitant COPD or CVD and those with COPD/CVD, the differences between the CLD and COPD/CVD groups were similar to the main analysis, although the difference for any healthcare barriers was not statistically significant (Table A5 in Supplement).

### Recurrent Acute Care Use

**Figure 4:**
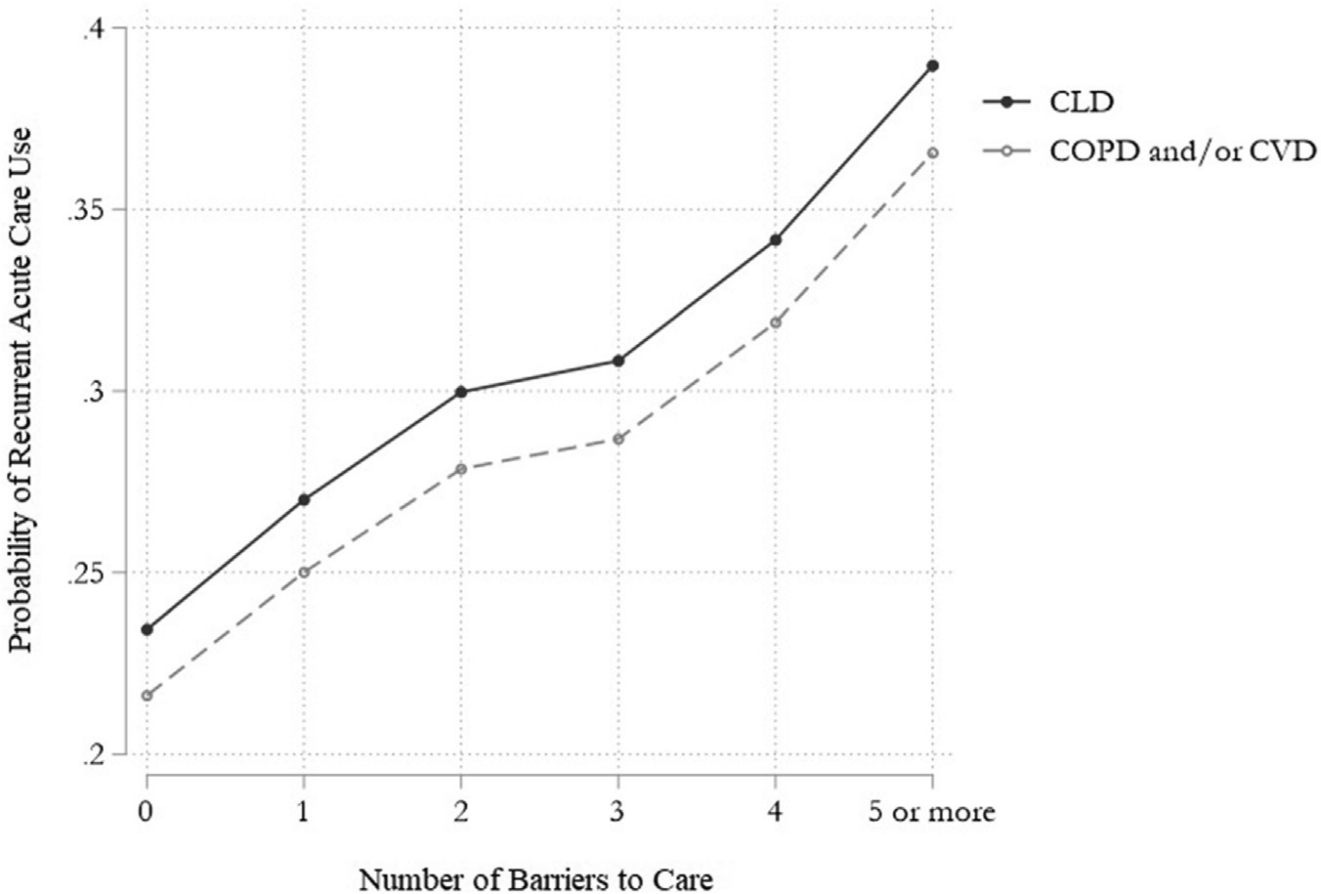
Adjusted probability of recurrent acute care use in the past year for adults with chronic liver disease compared to chronic obstructive pulmonary disease and/or cardiovascular disease, by number of barriers to care (n = 42,360). Source: National Health Interview Survey, 2011–2017. Predicted probabilities were obtained from a logistic regression model that adjusted for disease group, sex, age, race or ethnicity, comorbidity count, fair or poor health, functional limitation due to health, education, employment, living alone, household poverty, material hardship, insurance, US Census region, and year. All odds ratios were statistically significant (*P* < .001) in comparison with the no barriers (reference) group. COPD, chronic obstructive pulmonary disease; CLD, chronic liver disease; CVD, cardiovascular disease.

Recurrent hospitalizations and/or ED visits in the past year were more prevalent in the CLD than the COPD/CVD group (+5.3 pp; 95% CI, 3.6%–6.9%; *P* < .001) (Table 2). More respondents with recurrent acute care use had at least one healthcare barrier (Table A6 in Supplement). There was a dose-dependent relationship between the probability of recurrent acute care use and number of barriers with the highest adjusted probability of recurrent acute care use for those with at least 5 healthcare barriers and CLD (predicted probability, 0.37; 95% CI, 0.34–0.39) (Figure 4).

## Discussion

In this study, which is representative of over 43 million community-dwelling US adults with CLD or COPD/CVD, 3 main findings should be highlighted. First, we found that more adults with CLD experienced any healthcare barriers, including unaffordability, organizational, and transportation-related barriers compared to those with COPD/CVD. Second, the likelihood of any barriers, healthcare–related unaffordability, or organizational barriers within healthcare, were significantly higher for the CLD than COPD/CVD group after adjusting for differences in sociodemographic and health characteristics. Third, our study identified a positive association between the prevalence of healthcare barriers and the probability of recurrent acute care use for CLD and COPD/CVD.

Building on prior work,^11^^,^^16^, ^17^, ^18^, ^19^, ^20^, ^21^ this study distinguishes itself in several ways. First, while prior studies^18^, ^19^, ^20^, ^21^ have reported on specific hardships, including financial and transportation insecurity, among US adults with CLD compared to those without CLD, the current study includes a more comprehensive list of barriers throughout the process of seeking care in the community setting, including organizational barriers. This study demonstrates that the CLD population has a higher crude prevalence and probability of any self-reported healthcare barriers across the care continuum compared to individuals with COPD/CVD who share similar need for healthcare. Second, this study demonstrates a larger prevalence of socioeconomic vulnerabilities among the population with CLD compared to COPD/CVD, and the cumulative effect of different population characteristics, including socioeconomic factors, on the probability of experiencing healthcare barriers. Third, this study compares the relationship between the prevalence of healthcare barriers on recurrent acute care use in similarly complex medical conditions with high risk for hospital use (CLD vs COPD/CVD).

Our findings have important practice and policy implications. First, our findings highlight the discrepancy in prevalence and likelihood of any healthcare barriers between adults with CLD compared to those with COPD/CVD in the US, despite both chronic disease groups having comparably high morbidity, hospitalization, and mortality risks. These differences may be attributable to differences in the population characteristics. Our study demonstrated a greater proportion of socioeconomic vulnerabilities, including poverty, receipt of government insurance, and lack of health insurance, among adults with CLD compared to COPD/CVD. The cumulative effect of these socioeconomic risks constituted up to 42% of the difference in likelihood of any healthcare barriers for the CLD population. Similarly, these differences in covariates accounted for up to 51%, 33%, 30%, and 42% of the difference in likelihood of experiencing any healthcare–related financial unaffordability, any organizational barrier at the entry to healthcare, any organizational barrier within healthcare, and any healthcare–related transportation insecurity, respectively. While the adjusted analyses isolate the independent effect of the disease group on the likelihood of healthcare barriers, the unadjusted analyses provide information that can be more useful to providers to identify individuals with CLD who may be at higher risk for barriers to care. As there is increasing attention on the influence of social factors on health outcomes and a momentum towards the identification of social risks and health-related social needs in clinical settings,^35^, ^36^, ^37^ it is important to recognize that persons with CLD may be more socioeconomically vulnerable and at higher risk for healthcare barriers and hospital-based care compared to adults with COPD/CVD. Screening for health-related social needs and healthcare barriers among individuals with CLD could help identify potentially mutable factors that affect the risk of preventable acute care use.

Among the different types of healthcare barriers, organizational barriers within healthcare were the most salient barrier type that was significantly different between respondents with CLD compared to those with COPD/CVD after statistical adjustment for differences in sociodemographic, health, and insurance characteristics. This finding calls attention to health systems to proactively address mutable factors within their purview to prevent delays in receipt of medical care for persons with CLD.

Second, the stratified analyses further revealed where disparities may exist. Adults 65 years or older and Medicare enrollees with CLD had the largest difference in probability of any healthcare barriers compared to persons with COPD/CVD, which may be a function of Medicare’s unique role in the HRRP. While hospital readmission rates for those with target conditions in the setting of private insurance or Medicaid also decreased, the HRRP had the largest effect in the reduction of hospital readmission rates for Medicare beneficiaries.^38^ The HRRP incentivized providers and health systems to execute outpatient programs for targeted conditions like COPD and heart failure^39^, ^40^, ^41^ as the underuse of outpatient care was considered a mechanism by which preventable hospitalizations occurred.^42^ Therefore, the HRRP may have attenuated the probability of experiencing healthcare barriers for older adults or Medicare enrollees with COPD/CVD but not for those with CLD. Similar policies for persons with CLD have the potential to reduce preventable utilization of acute care through the mitigation of health-care barriers in the community setting.

Third, we recognize that the frequency of healthcare barriers is likely a function of one’s attempts to seek medical care; for example, if one cannot overcome organizational barriers at the point of entry (ie, establish care), one is less likely to encounter organizational barriers within the healthcare system as previously shown after Medicaid expansion.^43^ The lack of significant difference in the adjusted number of healthcare barriers between CLD and COPD/CVD could be explained by the greater proportion of individuals with organizational barriers at the point of entry in the CLD group. Had these respondents with organizational barriers at entry experienced the counterfactual, they may have experienced more organizational barriers within the healthcare system including transportation and financial barriers.

This study has several limitations. First, the study is a pooled cross-sectional observational study; therefore, findings are not interpretable as causal inferences. Findings are intended to be descriptive and to provide nationally representative estimates about the extent and probability of healthcare barriers among US adults with CLD compared to COPD/CVD. Second, there may be an underestimation of both disease populations because the survey questions used to identify CLD, COPD, and CVD assumed that the individual had been in contact with a doctor or other healthcare professional to obtain a diagnosis. Therefore, our findings are conditional on a prior encounter with a healthcare provider that diagnosed the respondent with a chronic condition. Our data may have also missed the most vulnerable persons as the NHIS does not survey hospitalized patients. Third, we were unable to delineate the etiology or severity of liver disease (eg, cirrhosis), using the available data, which may have implications on the type of care received by the respondent. Fourth, the data lacked information about rural or urban classification, which can affect differences in healthcare access.

In conclusion, findings from this nationally representative study revealed that US adults with CLD had a greater prevalence and likelihood of any healthcare barriers across the care seeking spectrum compared to adults with COPD/CVD. The higher burden of socioeconomic vulnerabilities in the CLD population contributes to its higher relative probability of healthcare barriers compared to the COPD/CVD population. A higher prevalence of healthcare barriers is associated with increased risk of potentially avoidable recurrent acute care use. This study highlights the need to consider CLD as a priority condition in future public policies and disease-specific programs such that resources can be appropriately directed to reduce the burden of socioeconomic vulnerabilities, barriers to care, and potentially avoidable recurrent acute care use in this disease population.
