# Supplementary material for: Prevalence of Healthcare Barriers Among US Adults With Chronic Liver Disease Compared to Other Chronic Diseases
Source: Gastro Hep Adv. 2024 May 17;3(6):796–808. doi: 10.1016/j.gastha.2024.05.004 (PMC11401582; doi:10.1016/j.gastha.2024.05.004)
Supplement: Supplementary Figures and Tables [file mmc1.docx]

**Supplemental Material**

**eMethods.**

**eReferences.**

**eFigure 1.** Study Population Flowchart

**eTable 1.** Unweighted Adjusted Incident Rate Ratios for Healthcare Barriers for Chronic Liver Disease vs. Chronic Obstructive Pulmonary Disease and/or Cardiovascular Disease and Goodness of Fit Measures Using Different Count Models

**eTable 2.** Sociodemographic and Health Characteristics by Healthcare Barriers for Chronic Liver Disease

**eTable 3.** Sociodemographic and Health Characteristics by Healthcare Barriers for Chronic Obstructive Pulmonary Disease and/or Cardiovascular Disease

**eTable 4.** Unadjusted and Adjusted Weighted Incident Rate Ratios Using Nested Hurdle Logit-Negative Binomial Regression Models to Assess the Relationship between Healthcare Barriers and Chronic Liver Disease vs. Chronic Obstructive Pulmonary Disease and/or Cardiovascular Disease

**eTable 5.** Sensitivity Analysis using Hurdle Logit-Negative Binomial Regression Model to Assess the Relationship between Healthcare Barriers and Disease Groups

**eTable 6.** Acute Care Utilization for Chronic Liver Disease and Chronic Obstructive Pulmonary Disease and/or Cardiovascular Disease, by Healthcare Barriers

**eMethods**

*Data source*

The National Health Interview Survey (NHIS) uses a complex, multistage sample design that provides analytical weight adjustments to yield nationally representative estimates.^1^ NHIS includes three main components, including the Family Core, Sample Adult Core, and Sample Child Core. From each randomly selected US household, information about the family, a sample adult, and a sample child, if available, are recorded in the Family, Sample Adult, and Sample Child Core, respectively. We used the Sample Adult Core for this study.

*Healthcare barriers*

We were interested in specific barriers, including self-reports about being declined as a new patient, having trouble establishing care with a doctor, having one’s health coverage declined, and needing but forgoing follow-up or specialty care due to costs. These specific barriers were captured by our data source from 2011 to 2017 and were no longer surveyed starting 2018.

We defined healthcare barriers as a *yes* response to questions about healthcare unaffordability, organizational barriers, and lack of transportation in the past year:

1. Was there any time when you needed medical care but did not get it because of the cost?
2. Was there any time when you needed any of the following, but didn’t get it because you couldn’t afford it …prescription medicines?
3. Was there any time when you needed any of the following, but didn’t get it because you couldn’t afford it …follow-up care?
4. Was there any time when you needed any of the following, but didn’t get it because you couldn’t afford it …to see a specialist?
5. Did you have any trouble finding a general doctor or provider who would see you?
6. Were you told by a doctor’s office or clinic that they would not accept you as a new patient?
7. Were you told by a doctor’s office or clinic that they did not accept your healthcare coverage?
8. Have you delayed getting care for any of the following reasons in the past 12 months? …You couldn’t get an appointment soon enough?
9. Have you delayed getting care for any of the following reasons in the past 12 months? …The clinic/ doctor’s office wasn’t open when you could get there?
10. Have you delayed getting care for any of the following reasons in the past 12 months? …You couldn’t get through on the telephone?
11. Have you delayed getting care for any of the following reasons in the past 12 months? …Once you get there, you have to wait too long to see the doctor?
12. Have you delayed getting care for any of the following reasons in the past 12 months? …You didn’t have transportation?

We also included any respondents who responded *no* to the following question:

1. Is there a place that you usually go to when you are sick or need advice about your health?

*Covariates*

Age group categories (18 to 34, 35 to 54, 55 to 64, 65 to 85 years) were selected based on increasing liver-related mortality and cirrhosis among persons 34 years or younger and more cirrhosis- and hepatocellular carcinoma-related deaths among persons at least 55 years old,^2^ which may be associated with differential healthcare access. We assessed for racial and ethnic differences using mutually exclusive racial and ethnic subgroups, including Non-Hispanic White, Hispanic, Non-Hispanic Black, Non-Hispanic Asian, Non-Hispanic American Indian or Alaskan Native, and Non-Hispanic Other, as similarly done in prior work.^3^ Health factors, including the number of comorbidities, functional limitation due to health, and fair or poor health status (vs. excellent, very good, or good health), were included because individuals with more complex healthcare needs and higher comorbidity burden are at risk of access and care coordination challenges.^4-5^ Education (less than high school graduate vs. high school graduate or higher) was assessed as a potential confounder given its established relationship with healthcare utilization and outcomes.^6^ Living alone as a surrogate for household isolation is included based on the higher likelihood of healthcare barriers among isolated adults.^7^ Competing personal priorities, including work and need for basic necessities, have been shown to contribute to delays in needed medical care.^8^ Consistent with prior studies,^9^ household poverty was defined as being above or below the federal poverty level (FPL) (<200% vs. ≥200% FPL), which was determined by comparing the reported total household income in the previous calendar year to the US Census Bureau’s poverty benchmarks for the year in question.^10^ We included insurance (uninsured, public insurance, Medicare, private insurance) based on previously reported differences in healthcare barriers by type of coverage.^11-12^

*Statistical analysis*

There were excess zeros in the distribution of the primary outcome in which 64.5% of the study population, 65.7% of the COPD/CVD group, and 55.3% of the CLD group had zero healthcare barriers. We compared count models, including Poisson, negative binomial regression (NBR), zero-inflated Poisson, zero-inflated NBR, hurdle logit-Poisson, and hurdle logit-NBR, using unweighted data which provided goodness of fit measures (AIC and BIC) and likelihood ratio tests (eTable 1). The hurdle logit-NBR model had the lowest AIC and BIC and a significant likelihood ratio test of alpha for overdispersion (eTable 1). We also selected the hurdle logit-NBR model based on its assumption that all zero counts were from one source in contrast to the zero-inflated NBR model which assumed that zero counts were from two processes, including one that assumed that a subgroup of individuals were not sampled or not at risk for the outcome.^13-14^ All respondents in our sample were asked the same set of questions about barriers to care; therefore, sampling zeros did not contribute toward the zero counts in this study.

We performed nested hurdle logit-NBR models that sequentially added covariates to the prior model and tested the model fit using the adjusted Wald test for our main analysis. We also assessed for multicollinearity among the covariates using the mean and individual variance inflation factor (VIF).^15^

The adjusted Wald test showed that each nested hurdle logit-NBR model with additional covariates was preferred over the prior model except for the one with employment (*P*=0.06). The mean VIF for employment was 1.47; therefore, significant correlation with the other independent covariates was not present. As we selected the employment variable *a priori* based on its hypothesized relationship with healthcare barriers as a competing personal priority, we included employment in our final multivariable regression model.

**eReferences**

1. National Center for Health Statistics. Centers for Disease Control and Prevention. National Health Interview Survey. Updated November 9, 2023. Accessed November 16, 2023. https://www.cdc.gov/nchs/nhis/index.htm
2. Tapper EB, Parikh ND. Mortality due to cirrhosis and liver cancer in the United States, 1999-2016: observation study. *BMJ* 2018;18(362):k2817. doi: 10.1136/bmj.k2817
3. Mahajan S, Caraballo C, Lu Yuan, et al. Trends in Differences in Health Status and Health Care Access and Affordability by Race and Ethnicity in the United States, 1999-2018. *JAMA* 2021;326(7):637-648. doi:10.1001/jama.2021.9907
4. Osborn R, Moulds D, Schneider EC, et al. Primary Care Physicians in Ten Countries Report Challenges Caring for Patients with Complex Health Needs. *Health Aff (Millwood)* 2015;34(12):2104-2112.
5. Bierman AS, Wang J, O’Malley PG, Moss DK. Transforming care for people with multiple chronic conditions: Agency for Healthcare Research and Quality’s research agenda. *Health Serv Res* 2021;56(Suppl 1):973-979.
6. Zajacova A and Lawrence EM. The relationship between education and health: reducing disparities through a contextual approach. *Annu Rev Public Health* 2018;39:273-289. doi: 10.1146/annurev-publhealth-031816-044628
7. Lewis C, Shah T, Abrams MK. Sick and Alone: High-Need, Socially Isolated Adults Have More Problems, but Less Support. The Commonwealth Fund. January 12, 2018. Accessed November 16, 2023. https://www.commonwealthfund.org/blog/2018/sick-and-alone-high-need-socially-isolated-adults-have-more-problems-less-support
8. Diamant AL, Hays RD, Morales LS, et al. Delays and unmet need for health care among adult primary care patients in a restructured urban public health system. *Am J Public Health* 2004;94(5):783-789. doi: 10.2105/ajph.94.5.783
9. Caraballo C, Ndumele CD, Roy B, et al. Trends in Racial and Ethnic Disparities in Barriers to Timely Medical Care Among Adults in the US, 1999 to 2018. *JAMA Health Forum* 2022;3(10):e223856. doi:10.1001/jamahealthforum.2022.3856
10. Blewett LA, Rivera Drew JA, King ML, Williams KCW, Del Ponte N, Convey P. IPUMS Health Surveys: National Health Interview Survey, Version 7.1[dataset]. Minneapolis, MN: IPUMS, 2021. Accessed August 22, 2023. https://doi.org/10.18128/D070.V7.1
11. Allen EM, Call KT, Beebe TJ, McAlpine DD, Johnson PJ. Barriers to Care and Health Care Utilization Among the Publicly Insured. *Med Care* 2017;55(3):207-214. doi: 10.1097/MLR.0000000000000644
12. Wray CM, Khare M, Keyhani S. *JAMA Netw Open* 2021;4(6):e2110275. doi:10.1001/jamanetworkopen.2021.10275
13. Feng, CX A comparison of zero-inflated and hurdle models for modeling zero-inflated count data. *J Stat Distrib App*. 2021;8:1-19. doi: https://doi.org/10.1186/s40488-021-00121-4
14. Long JS, Freese J. Chapter 7: Models for Count Outcomes. In: Long JS, Freese J. *Regression Models for Categorical Dependent Variables Using Stata*. Stata Press;2001:223-260.
15. Liao D, Valliant R. Variance inflation factors in the analysis of complex survey data. *Survey Methodology.* 2012;38(1):53-62.

**eFigure 1.** Study Population Flowchart

232,235 adults included in the National Health Interview Survey from 2011 to 2017

28 adults excluded due to missing information about having CLD, COPD, or CVD

232,207 adults with complete information about CLD, COPD, and CVD

185,170 adults excluded for not having CLD, COPD, or CVD

47,037 adults in the study population with CLD or COPD and/or CVD

5,062 adults with CLD

41,975 adults with COPD and/or CVD

Abbreviations: CLD, chronic liver disease; COPD, chronic obstructive pulmonary disease; CVD, cardiovascular disease

The COPD and/or CVD (non-CLD) group included 32,578 respondents with CVD, 15,144 with COPD, and 5,747 with both CVD and COPD.

**eTable 1.** Unweighted Adjusted Incident Rate Ratios for Healthcare Barriers for Chronic Liver Disease vs. Chronic Obstructive Pulmonary Disease and/or Cardiovascular Disease and Goodness of Fit Measures Using Different Count Models (n=42,370)

|  | Model | Any Barriers | | | Number of Barriers | | | Goodness of Fit Measures | | |
| --- | --- | --- | --- | --- | --- | --- | --- | --- | --- | --- |
|  |  | **IRR** | **95% CI** | ***P*-value** | **IRR** | **95% CI** | ***P*-value** | **AIC** | **BIC** | ***P*-value of LRT of Alpha** |
| A | Poisson | -- | -- | -- | 1.10 | 1.07-1.13 | <.001 | 111843.7 | 112120.7 | N/A |
| B | Negative binomial | -- | -- | -- | 1.12 | 1.07-1.18 | <.001 | 98565.42 | 98851.01 | <.001 |
| C | Zero-inflated Poisson | 1.15 | 1.06-1.25 | 0.001 | 1.05 | 1.02-1.09 | 0.003 | 99582.66 | 100136.5 | N/A |
| D | Zero-inflated negative binomial | 1.22 | 1.07-1.40 | 0.003 | 1.05 | 1.00-1.11 | 0.043 | 97128.88 | 97691.4 | <.001 |
| E | Hurdle logit-Poisson | 1.16 | 1.08-1.24 | <.001 | 1.05 | 1.02-1.09 | 0.002 | 99586.83 | 100140.7 | N/A |
| F | Hurdle logit-negative binomial | 1.16 | 1.08-1.24 | <.001 | 1.06 | 1.01-1.12 | 0.026 | 97117.73 | 97680.25 | <.001 |

Source: National Health Interview Survey, 2011-2017

Abbreviations: IRR, incident rate ratio; AIC, Akaike Information Criterion; BIC, Bayesian Information Criterion; LRT, likelihood ratio test

All models use an unweighted sample of 42,370 respondents with CLD or COPD/CVD and adjusts for sex, age, race or ethnicity, comorbidity count, fair or poor health, functional limitation due to health, education, unemployment, living alone, poverty, receipt of government support, health insurance, US Census region, and survey year.

IRR for any and number of barriers are available only for two-part models including zero-inflated Poisson, zero-inflated negative binomial, hurdle logit-Poisson, and hurdle logit-negative binomial regression.

**eTable 2.** Sociodemographic and Health Characteristics by Healthcare Barriers for Chronic Liver Disease (n=5,062)

| Characteristic  Respondents, unweighted no. | Zero Barriers | ≥ 1 Barriers | *P* value |
| --- | --- | --- | --- |
|  | 2,800 | 2,262 |  |
| Age groups, y^a^ |  |  |  |
| 18-34 | 11.8 (10.1-13.6) | 15.1 (13.2-17.2) | <.001 |
| 35-54 | 30.9 (28.7-33.3) | 41.0 (38.3-43.8) |  |
| 55-64 | 28.8 (26.6-31.1) | 28.8 (26.5-31.2) |  |
| 65-85 | 28.5 (26.4-30.8) | 15.1 (13.3-17.1) |  |
| Female sex | 49.4 (46.9-51.9) | 54.0 (51.2-56.8) | 0.02 |
| Race or ethnicity^b^ |  |  |  |
| White | 66.5 (64.0-68.9) | 64.9 (62.3-67.4) | 0.002 |
| Black or African American | 8.3 (7.0-9.7) | 8.5 (7.2-9.9) |  |
| Hispanic | 16.1 (14.1-18.3) | 19.2 (17.2-21.5) |  |
| Asian | 6.2 (5.1-7.6) | 3.5 (2.7-4.6) |  |
| American Indian or Alaska Native | 1.0 (0.6-1.6) | 1.1 (0.7-1.7) |  |
| Other | 1.9 (1.3-2.7) | 2.8 (2.0-4.0) |  |
| Comorbidity burden |  |  |  |
| Number of comorbidities,  median (range)^c^ | 3 (1-10) | 4 (1-10) | <.001 |
| Functional limitation due to health^d^ (n=5,056) | 62.6 (60.2-64.8) | 75.1 (72.5-77.5) | <.001 |
| Fair or poor health^e^ (n=5,056) | 33.5 (31.2-35.8) | 51.3 (48.6-53.9) | <.001 |
| Education attainment (n=5,036) |  |  |  |
| Less than high school graduate level | 6.6 (5.5-7.9) | 7.9 (6.6-9.5) | 0.15 |
| Employment (n=5,052) |  |  |  |
| Currently unemployed | 56.2 (53.7-58.7) | 59.7 (57.1-62.3) | 0.06 |
| Living alone | 21.4 (19.8-23.0) | 25.5 (23.6-27.6) | 0.002 |
| Household income^f^ (n=4,791) |  |  |  |
| Below poverty threshold | 14.5 (13.0-16.2) | 26.8 (24.6-29.1) | <.001 |
| Receipt of any government support^g^ | 21.8 (19.9-23.8) | 39.0 (36.4-41.6) | <.001 |
| Income support^h^ (n=5,058) | 9.9 (8.6-11.4) | 12.6 (11.1-14.2) | 0.009 |
| Rent assistance^i^ (n=5,060) | 5.7 (4.8-6.7) | 7.1 (6.0-8.3) | 0.06 |
| Food support^j^ (n=5,058) | 17.2 (15.5-19.0) | 33.4 (30.9-36.0) | <.001 |
| Other welfare^k^ (n=5,056) | 0.9 (0.6-1.3) | 2.7 (2.0-3.6) | <.001 |
| US region |  |  |  |
| Northeast | 18.7 (16.7-20.9) | 13.5 (11.8-15.5) | 0.003 |
| North Central/ Midwest | 19.5 (17.6-21.5) | 20.3 (17.9-22.9) |  |
| South | 34.6 (32.3-37.0) | 38.0 (35.2-40.8) |  |
| West | 27.2 (25.0-29.5) | 28.3 (25.7-31.0) |  |
| Insurance coverage^l^ (n=4,924) |  |  |  |
| None | 4.6 (3.6-5.8) | 19.3 (17.2-21.6) | <.001 |
| Public insurance | 21.6 (19.5-23.8) | 26.0 (23.6-28.6) |  |
| Medicare | 16.2 (14.4-18.1) | 14.1 (12.3-16.2) |  |
| Private insurance | 57.7 (55.0-60.2) | 40.6 (37.6-43.6) |  |

Source: National Health Interview Survey, 2011-2017

Abbreviations: CVD, cardiovascular disease; CLD, chronic liver disease; COPD, chronic obstructive pulmonary disease; SSI, supplemental security income; SNAP, Supplemental Nutrition Assistance Program; WIC, Special Supplemental Nutrition Program for Women, Infants, and Children

Data are reported as percent values with 95% confidence intervals unless otherwise indicated.

Denominators per disease category are reported as unweighted observations.

Weighted estimates were obtained using *svy*. Categorical variables were compared using Chi-squared tests. The number of comorbidities was compared using an adjusted Wald test and the median (range) were obtained using the sampling weight as an analytic weight.

^a^ Age groups include the following years: 18-34, 35-55, 56-64, and 65-85 years old.

^b^ Race or ethnicity includes self-reported Non-Hispanic White (*White*), Non-Hispanic Black (*Black or African-American)*, Hispanic (*Hispanic*), Non-Hispanic Asian (*Asian*), Non-Hispanic American Indian or Alaska Native (*American Indian or Alaska Native*), or Non-Hispanic Other (*Other*) which includes multiple race and race groups that are not releasable.

^c^ Comorbidity count includes self-reported arthritis, asthma, BMI ≥ 30, cancer, CVD, chronic liver disease, COPD, diabetes, hypertension, and kidney disease.

^d^ Functional limitation due to health includes responses about having any functional difficulty because of a health problem.

^e^ Fair or poor health includes responses about self-reported health status and is compared to those with excellent, very good, or good health.

^f^ Poverty threshold is based on family size, number of children under 18 years old, and reported before-tax combined money income from all sources, excluding noncash benefits, during the preceding calendar year as compared to the U.S. Census Bureau’s poverty thresholds for the preceding calendar year. The household income category of *Below poverty threshold* includes persons who report household income below the poverty threshold (vs. at or above poverty threshold).

^g^ Receipt of any government support includes receiving any support for income (SSI and/or cash assistance), housing (rent assistance), food (food stamps, SNAP, and/or WIC), and/or other welfare (assistance with getting a job, placement in education or job training programs, transportation, or childcare) in the previous calendar year.

^h^ Income support includes responses about receiving income from SSI and/or cash assistance in the previous calendar year.

^i^ Rent assistance includes responses about having received public rent assistance.

^j^ Food support includes responses about receiving any food stamps, SNAP benefits, and/or WIC.

^k^ Other welfare includes assistance with getting a job, placement in education or job training programs, transportation, or childcare).

^l^ Insurance category *None* includes persons without any insurance coverage including having only single service plans, category *Public insurance* includes any public insurance via Medicaid, other state or local government program, Children’s Health Insurance Program, or Medicare for dual enrollees, category *Medicare* includes Medicare only beneficiaries, and category *Private insurance* includes enrollment in any private insurance.

**eTable 3.** Sociodemographic and Health Characteristics by Healthcare Barriers for Chronic Obstructive Pulmonary Disease and/or Cardiovascular Disease (n=41,975)

| Characteristic  Respondents, unweighted no. | Zero Barriers | ≥ 1 Barriers | *P* value |
| --- | --- | --- | --- |
|  | 27,557 | 14,418 |  |
| Age groups, y^a^ |  |  |  |
| 18-34 | 7.7 (7.2-8.3) | 15.9 (14.9-16.8) | <.001 |
| 35-54 | 19.3 (18.7-20.0) | 31.6 (30.6-32.7) |  |
| 55-64 | 20.6 (20.0-21.4) | 25.2 (24.2-26.2) |  |
| 65-85 | 52.3 (51.4-53.2) | 27.4 (26.4-28.4) |  |
| Female sex | 49.7 (48.9-50.6) | 55.7 (54.6-56.8) | <.001 |
| Race or ethnicity^b^ |  |  |  |
| White | 78.3 (77.5-79.1) | 69.6 (68.4-70.7) | <.001 |
| Black or African American | 9.8 (9.3-10.4) | 13.6 (12.8-14.5) |  |
| Hispanic | 7.3 (6.8-7.9) | 11.0 (10.3-11.8) |  |
| Asian | 2.6 (2.4-2.9) | 2.7 (2.3-3.1) |  |
| American Indian or Alaska Native | 0.6 (0.5-0.8) | 0.8 (0.6-1.0) |  |
| Other | 1.3 (1.2-1.5) | 2.4 (2.1-2.7) |  |
| Comorbidity burden |  |  |  |
| Number of comorbidities,  median (range)^c^ | 3 (1-9) | 3 (1-9) | <.001 |
| Functional limitation due to health^d^ (n=41,877) | 64.9 (64.0-65.7) | 74.3 (73.2-75.3) | <.001 |
| Fair or poor health^e^ (n=41,953) | 28.9 (28.2-29.7) | 41.7 (40.6-42.8) | <.001 |
| Education attainment (n=41,756) |  |  |  |
| Less than high school graduate level | 6.4 (5.9-6.8) | 6.7 (6.2-7.3) | 0.22 |
| Employment (n=41,898) |  |  |  |
| Currently unemployed | 65.2 (64.4-66.1) | 61.3 (60.1-62.4) | <.001 |
| Living alone | 23.9 (23.3-24.6) | 25.9 (25.1-26.7) | <.001 |
| Household income^f^ (n=38,825) |  |  |  |
| Below poverty threshold | 11.3 (10.8-11.8) | 22.6 (21.6-23.6) | <.001 |
| Receipt of any government support^g^ | 15.7 (15.0-16.4) | 32.5 (31.4-33.7) | <.001 |
| Income support^h^ (n=41,891) | 5.8 (5.4-6.2) | 9.5 (8.9-10.1) | <.001 |
| Rent assistance^i^ (n=41,891) | 4.5 (4.1-4.9) | 7.0 (6.4-7.6) | <.001 |
| Food support^j^ (n=41,918) | 12.3 (11.7-12.9) | 27.7 (26.6-28.8) | <.001 |
| Other welfare^k^ (n=5,056) | 0.6 (0.5-0.7) | 1.6 (1.3-1.8) | <.001 |
| US region |  |  |  |
| Northeast | 18.9 (17.9-19.8) | 13.7 (12.8-14.6) | <.001 |
| North Central/ Midwest | 25.0 (24.1-25.9) | 24.4 (23.1-25.8) |  |
| South | 37.6 (36.5-38.7) | 41.2 (39.7-42.6) |  |
| West | 18.5 (17.7-19.4) | 20.7 (19.6-21.9) |  |
| Insurance coverage^a^ (n=41,110) |  |  |  |
| None | 2.7 (2.4-3.0) | 17.9 (17.0-18.9) | <.001 |
| Public insurance | 13.1 (12.5-13.7) | 20.7 (19.8-21.7) |  |
| Medicare | 24.0 (23.2-24.7) | 18.4 (17.5-19.3) |  |
| Private insurance | 60.3 (59.4-61.2) | 43.0 (41.7-44.2) |  |

Source: National Health Interview Survey, 2011-2017

Abbreviations: CVD, cardiovascular disease; CLD, chronic liver disease; COPD, chronic obstructive pulmonary disease; SSI, supplemental security income; SNAP, Supplemental Nutrition Assistance Program; WIC, Special Supplemental Nutrition Program for Women, Infants, and Children

Data are reported as percent values with 95% confidence intervals unless otherwise indicated.

Denominators per disease category are reported as unweighted observations.

Weighted estimates were obtained using *svy*. Categorical variables were compared using Chi-squared tests. The number of comorbidities was compared using an adjusted Wald test and the median (range) were obtained using the sampling weight as an analytic weight.

^a^ Age groups include the following years: 18-34, 35-55, 56-64, and 65-85 years old.

^b^ Race or ethnicity includes self-reported Non-Hispanic White (*White*), Non-Hispanic Black (*Black or African-American)*, Hispanic (*Hispanic*), Non-Hispanic Asian (*Asian*), Non-Hispanic American Indian or Alaska Native (*American Indian or Alaska Native*), or Non-Hispanic Other (*Other*) which includes multiple race and race groups that are not releasable.

^c^ Comorbidity count includes self-reported arthritis, asthma, BMI ≥ 30, cancer, CVD, chronic liver disease, COPD, diabetes, hypertension, and kidney disease.

^d^ Functional limitation due to health includes responses about having any functional difficulty because of a health problem.

^e^ Fair or poor health includes responses about self-reported health status and is compared to those with excellent, very good, or good health.

^f^ Poverty threshold is based on family size, number of children under 18 years old, and reported before-tax combined money income from all sources, excluding noncash benefits, during the preceding calendar year as compared to the U.S. Census Bureau’s poverty thresholds for the preceding calendar year. The household income category of *Below poverty threshold* includes persons who report household income below the poverty threshold (vs. at or above poverty threshold).

^g^ Receipt of any government support includes receiving any support for income (SSI and/or cash assistance), housing (rent assistance), food (food stamps, SNAP, and/or WIC), and/or other welfare (assistance with getting a job, placement in education or job training programs, transportation, or childcare) in the previous calendar year.

^h^ Income support includes responses about receiving income from SSI and/or cash assistance in the previous calendar year.

^i^ Rent assistance includes responses about having received public rent assistance.

^j^ Food support includes responses about receiving any food stamps, SNAP benefits, and/or WIC.

^k^ Other welfare includes assistance with getting a job, placement in education or job training programs, transportation, or childcare).

^l^ Insurance category *None* includes persons without any insurance coverage including having only single service plans, category *Public insurance* includes any public insurance via Medicaid, other state or local government program, Children’s Health Insurance Program, or Medicare for dual enrollees, category *Medicare* includes Medicare only beneficiaries, and category *Private insurance* includes enrollment in any private insurance.

**eTable 4.** Unadjusted and Adjusted Weighted Incident Rate Ratios Using Nested Hurdle Logit-Negative Binomial Regression Models to Assess the Relationship between Healthcare Barriers and Chronic Liver Disease vs. Chronic Obstructive Pulmonary Disease and/or Cardiovascular Disease

| Nested Model | Any Barriers | | | | Number of Barriers | | | | Adjusted Wald Test | Mean VIF |
| --- | --- | --- | --- | --- | --- | --- | --- | --- | --- | --- |
|  | **IRR** | **SE** | **95% CI** | ***P*-value** | **IRR** | **SE** | **95% CI** | ***P*-value** |  |  |
| A. Disease group | 1.54 | 0.02 | 1.43-1.67 | <.001 | 1.22 | 0.03 | 1.16-1.28 | <.001 | -- | -- |
| B. Same as model A plus sex | 1.55 | 0.02 | 1.44-1.67 | <.001 | 1.22 | 0.02 | 1.17-1.28 | <.001 | <.001 | 1.00 |
| C. Same as model B plus age | 1.27 | 0.04 | 1.16-1.40 | <.001 | 1.14 | 0.02 | 1.09-1.20 | <.001 | <.001 | 1.09 |
| D. Same as model C plus race or ethnicity | 1.25 | 0.04 | 1.14-1.37 | <.001 | 1.14 | 0.04 | 1.08-1.20 | <.001 | <.001 | 1.06 |
| E. Same as model D plus comorbidity | 1.15 | 0.04 | 1.05-1.25 | 0.003 | 1.08 | 0.03 | 1.01-1.14 | 0.01 | <.001 | 1.08 |
| F. Same as model E plus fair or poor health | 1.13 | 0.04 | 1.03-1.25 | 0.01 | 1.07 | 0.03 | 1.01-1.13 | 0.03 | <.001 | 1.10 |
| G. Same as model F plus functional limitation due to health | 1.14 | 0.05 | 1.03-1.27 | 0.01 | 1.07 | 0.03 | 1.02-1.13 | 0.01 | <.001 | 1.13 |
| H. Same as model G plus education | 1.12 | 0.05 | 1.01-1.25 | 0.03 | 1.07 | 0.03 | 1.01-1.13 | 0.01 | <.001 | 1.14 |
| I. Same as model H plus unemployment | 1.12 | 0.05 | 1.01-1.24 | 0.03 | 1.07 | 0.03 | 1.02-1.13 | 0.01 | 0.06 | 1.19* |
| J. Same as model I plus living alone | 1.11 | 0.05 | 1.00-1.24 | 0.05 | 1.07 | 0.03 | 1.01-1.12 | 0.01 | <.001 | 1.19 |
| K. Same as model J plus poverty | 1.12 | 0.05 | 1.01-1.24 | 0.04 | 1.07 | 0.03 | 1.01-1.12 | 0.02 | <.001 | 1.22 |
| L. Same as model K plus receipt of government support | 1.11 | 0.05 | 1.00-1.24 | 0.06 | 1.06 | 0.03 | 1.01-1.12 | 0.02 | <.001 | 1.26 |
| M. Same as model L plus health insurance | 1.13 | 0.05 | 1.02-1.26 | 0.02 | 1.06 | 0.03 | 1.01-1.12 | 0.02 | <.001 | 1.34 |
| N. Same as model M plus US Census region | 1.12 | 0.05 | 1.01-1.25 | 0.03 | 1.05 | 0.03 | 1.00-1.11 | 0.06 | <.001 | 1.42 |
| O. Same as model N plus survey year | 1.12 | 0.05 | 1.01-1.24 | 0.03 | 1.05 | 0.03 | 1.00-1.11 | 0.06 | 0.01 | 1.40 |

Source: National Health Interview Survey, 2011-2017

Abbreviations: IRR, incident rate ratio; SE, standard error; VIF, variance inflation factor; CLD, chronic liver disease; COPD, chronic obstructive pulmonary disease; CVD, cardiovascular disease

Weighted estimates were obtained using probability weights (pweight) and clustering at the primary sampling units.

Model A compares 47,037 respondents by disease groups of interest CLD vs. COPD and/or CVD (without CLD).

Model B compares 47,037 respondents by disease groups (CLD vs. COPD and/or CVD) and adjusts for biological sex.

Model C compares 47,037 respondents by disease groups and adjusts for biological sex and age (65 years or older vs. 18-34, 35-54, 55-64 years old).

Model D compares 47,037 respondents by disease groups and adjusts for biological sex, age, and race or ethnicity (White vs. Black, Hispanic, Asian, American Indian/ Alaskan Native, Other).

Model E compares 47,037 respondents by disease groups and adjusts for biological sex, age, race or ethnicity, and comorbidity (number of comorbidities).

Model F compares 47,009 respondents by disease groups and adjusts for biological sex, age, race or ethnicity, comorbidity, and fair or poor health.

Model G compares 46,905 respondents by disease groups and adjusts for biological sex, age, race or ethnicity, and comorbidity, fair or poor health, and functional limitation due to health.

Model H compares 46,663 respondents by disease groups and adjusts for biological sex, age, race or ethnicity, and comorbidity, fair or poor health, functional limitation due to health, and less than high school graduate level education. * Individual VIF for unemployment is 1.47.

Model I compares 46,589 respondents by disease groups and adjusts for biological sex, age, race or ethnicity, and comorbidity, fair or poor health, functional limitation due to health, less than high school graduate level education, and unemployment.

Model J compares 46,589 respondents by disease groups and adjusts for biological sex, age, race or ethnicity, and comorbidity, fair or poor health, functional limitation due to health, less than high school graduate level education, unemployment, and living alone.

Model K compares 43,289 respondents by disease groups and adjusts for biological sex, age, race or ethnicity, and comorbidity, fair or poor health, functional limitation due to health, less than high school graduate level education, unemployment, living alone, and poverty (household income below federal poverty level).

Model L compares 43,289 respondents by disease groups and adjusts for biological sex, age, race or ethnicity, and comorbidity burden, fair or poor health, functional limitation due to health, less than high school graduate level education, unemployment, living alone, poverty, and receipt of government support (receipt of government subsidy for income, food, other welfare).

Model M compares 42,370 respondents by disease groups and adjusts for biological sex, age, race or ethnicity, and comorbidity burden, fair or poor health, functional limitation due to health, less than high school graduate level education, unemployment, living alone, poverty, receipt of government support, and health insurance (private vs. no insurance, public, Medicare).

Model N compares 42,370 respondents by disease groups and adjusts for biological sex, age, race or ethnicity, and comorbidity burden, fair or poor health, functional limitation due to health, less than high school graduate level education, unemployment, living alone, poverty, receipt of government support, health insurance, and US Census region (Northeast vs. Midwest, South, West).

Model O compares 42,370 respondents by disease groups and adjusts for biological sex, age, race or ethnicity, and comorbidity burden, fair or poor health, functional limitation due to health, less than high school graduate level education, unemployment, living alone, poverty, receipt of government support, health insurance, US Census region, and survey year (2011-2013 vs. 2014-2017).

**eTable 5.** Sensitivity Analysis using Hurdle Logit-Negative Binomial Regression Model to Assess the Relationship between Healthcare Barriers and Disease Groups

| Disease Group | Any Barriers | | | | Number of Barriers | | | |
| --- | --- | --- | --- | --- | --- | --- | --- | --- |
|  | **IRR** | **SE** | **95% CI** | ***P*-value** | **IRR** | **SE** | **95% CI** | ***P*-value** |
| A. CLD vs. COPD and/or CVD | 1.12 | 0.05 | 1.01-1.24 | 0.03 | 1.05 | 0.03 | 1.00-2.71 | 0.06 |
| B. CLD only vs. COPD and/or CVD | 1.10 | 0.08 | 0.92-1.30 | 0.29 | 1.04 | 0.04 | 0.96-1.13 | 0.32 |

Source: National Health Interview Survey, 2011-2017

Abbreviations: IRR, incident rate ratio; SE, standard error; CLD, chronic liver disease; COPD, chronic obstructive pulmonary disease; CVD, cardiovascular disease

Model A is the primary analysis, which compares 42,370 respondents with CLD vs. COPD and/or CVD and adjusts for sex, age, race or ethnicity, comorbidity count, fair or poor health, functional limitation due to health, education, unemployment, living alone, poverty, receipt of government support, insurance type, US Census region, and survey year.

Model B replicates the primary analysis using the same covariates and compares 40,483 respondents with CLD only vs. COPD and/or CVD.

**eTable 6.** Acute Care Utilization for Chronic Liver Disease and Chronic Obstructive Pulmonary Disease and/or Cardiovascular Disease, by Healthcare Barriers

| Disease Group | Characteristic | Zero Barriers | ≥ 1 Barriers | *P* value |
| --- | --- | --- | --- | --- |
| Chronic Liver Disease | Acute care use in the past year (n=5,061) |  |  |  |
|  | ≥ 2 ED or hospital admissions | 24.4 (22.4-26.5) | 35.2 (32.8-37.7) | <.001 |
|  | ≥ 2 hospital admissions (n=5,055) | 8.1 (7.0-9.4) | 12.1 (10.5-13.9) | <.001 |
|  | ≥ 2 ED visits (n=5,011) | 15.5 (13.8-17.4) | 28.3 (26.1-30.7) | <.001 |
| Chronic Obstructive Pulmonary Disease and/or Cardiovascular Disease | Acute care use in the past year (n=41,962) |  |  |  |
|  | ≥ 2 ED or hospital admissions | 20.3 (19.7-21.0) | 30.9 (29.9-31.9) | <.001 |
|  | ≥ 2 hospital admissions (n=41,886) | 6.9 (6.5-7.3) | 9.0 (8.4-9.6) | <.001 |
|  | ≥ 2 ED visits (n=41,405) | 11.7 (11.2-12.2) | 22.9 (22.0-23.8) | <.001 |

Source: National Health Interview Survey, 2011-2017

Abbreviations: ED, emergency department

Data are reported as percent values with 95% confidence intervals.

Denominators per disease category are reported as unweighted observations.

Weighted proportions were obtained using *svy*. Categorical variables were compared using Chi-squared tests
